# Efficacy of Laser-Activated Irrigation Versus Ultrasonic-Activated Irrigation: A Systematic Review

**DOI:** 10.7759/cureus.36352

**Published:** 2023-03-19

**Authors:** Vijetha Badami, Sneha Akarapu, Hemabhanu Kethineni, Satya Priya Mittapalli, Kasi Reddy Bala, Syeda Farha Fatima

**Affiliations:** 1 Department of Conservative Dentistry and Endodontics, MNR Dental College and Hospital, Hyderabad, IND

**Keywords:** laser-activated irrigation, ultrasonic-activated irrigation, dentin debris removal, smear layer removal, antimicrobial efficacy

## Abstract

This study aimed to conduct a systematic review and critical analysis of the evidence pertaining to the efficacy of laser-activated irrigation (LAI) versus ultrasonic-activated irrigation (UAI) in mature permanent teeth. A comprehensive literature search was performed using PubMed and Google Scholar. Additionally, a hand search was performed to identify relevant studies related to UAI and LAI. The search covered all articles published from January 1997 to December 2021. The identified studies were screened for eligibility using the inclusion and exclusion criteria. The included articles were then subjected to data extraction and analysis. The search yielded 1,637 results. Of these, 23 articles were included in this systematic review. All included articles were assessed for the outcomes of antimicrobial efficacy, smear layer, and dentin debris removal. The majority of the articles reported the superiority of LAI over UAI. Within the confines of this systematic review, the current evidence mandates that LAI has superior efficacy over UAI in the elimination of microorganisms, dentin debris, and smear layer from the root canal system.

## Introduction and background

Endodontic treatment consists of thorough cleaning and disinfection of the root canal system to remove debris, microbial loads, and necrotic pulp tissue. Currently, mechanical instruments and disinfecting irrigants are used for this purpose [1].

When the root canal system is instrumented, dentin debris and an accumulated smear layer cover the canal walls [2]. The smear layer is a non-uniform, amorphous layer consisting of organic and inorganic components such as pathogenic organisms, their by-products, and parts of the odontoblastic process [3]. The smear layer has been shown to prevent both irrigants and sealants from penetrating the dentinal tubules [4]. This prevents proper cleaning and root canal filling. Consequently, chemical disinfection by irrigation is essential [5].

A syringe and a needle are frequently used in conjunction to irrigate the root canal. However, its effectiveness is constrained because the irrigant can only flow 1 mm past the tip of the needle [6,7]. This suggests that the irrigant frequently misses the apical region of the canal [8]. This encourages the continuation of biofilm and the survival of a large number of microflora, even after the chemomechanical preparation is deemed to be finished [9].

Additionally, *Enterococcus faecalis* and *Porphyromonas gingivalis* species have a 500-micron penetration limit in dentinal tubules and are the main causes of persistent periradicular pathosis [10]. Therefore, effective debridement and disinfection depend on the ability of the irrigant to penetrate sufficiently, especially in the untreated portions of the root canal [9].

To circumvent the disadvantages of syringe-needle irrigation, several more sophisticated techniques have been developed, including the use of ultrasonics and lasers. These are of utmost importance because they increase the effectiveness of irrigants [11-13]. Lasers can eliminate *Candida albicans* and highly resistant *E. faecalis* species in addition to cleaning and sterilizing the root canal dentin [14-16]. Laser-guided irrigation effectively removes the debris and smear layer from the root canal system by creating unstable vapor bubbles with a secondary cavitation effect [17]. The phenomena of cavitation and acoustic flow are produced by ultrasonic-guided irrigation and are beneficial for the more effective eradication of biofilm [18]. Ultrasonic-stimulated irrigation has been shown to remove more debris and smear layer than conventional irrigation [19].

Previously published systematic reviews have examined the cleaning and disinfection capabilities of ultrasonic-activated irrigation (UAI) and the disinfection efficacy of various laser applications [20,21]. None of the systematic reviews attempted to compare the efficacy of laser-activated irrigation (LAI) and UAI. Hence, this study aimed to conduct a systematic review and critical analysis of the evidence pertaining to the efficacy of LAI versus UAI in mature permanent teeth.

## Review

Methodology

Study Design

This systematic review is structured and adheres to the Preferred Reporting Items for Systematic Reviews and Meta-Analyses (PRISMA) standards [22]. The use of checklists in PRISMA is likely to raise reporting requirements for systematic reviews and provide transparency in the selection of papers for a systematic review.

Focused Research Question

The clinical question was formulated according to PICOS, and was as follows: “Which irrigation activation method, between the laser-activated irrigation and ultrasonic-activated irrigation, is more effective in terms of root canal disinfection, smear layer removal, debris removal, or cleanliness in human-extracted teeth?.” The population (P) considered here were mature permanent extracted teeth; the intervention (I) was irrigant activation methods; the comparison (C) was between UAI and LAI methods; the outcome (O) assessed were root canal disinfection, smear layer removal, debris removal, or cleanliness in human extracted teeth; and the study design (S) considered were all in vitro studies.

Literature Search

To find all relevant articles pertaining to UAI and LAI, a thorough literature search was conducted utilizing two electronic databases and a manual search. PubMed and Google Scholar were consulted by using the following search strategy: “(ultrasonic-activated irrigation) AND (laser-activated irrigation) AND (antimicrobial efficacy) OR (biofilm removal) OR (smear layer removal) OR (cleanliness) OR (debris removal).” All articles published from January 1997 to December 2021 were included in the search process.

**Figure 1:**
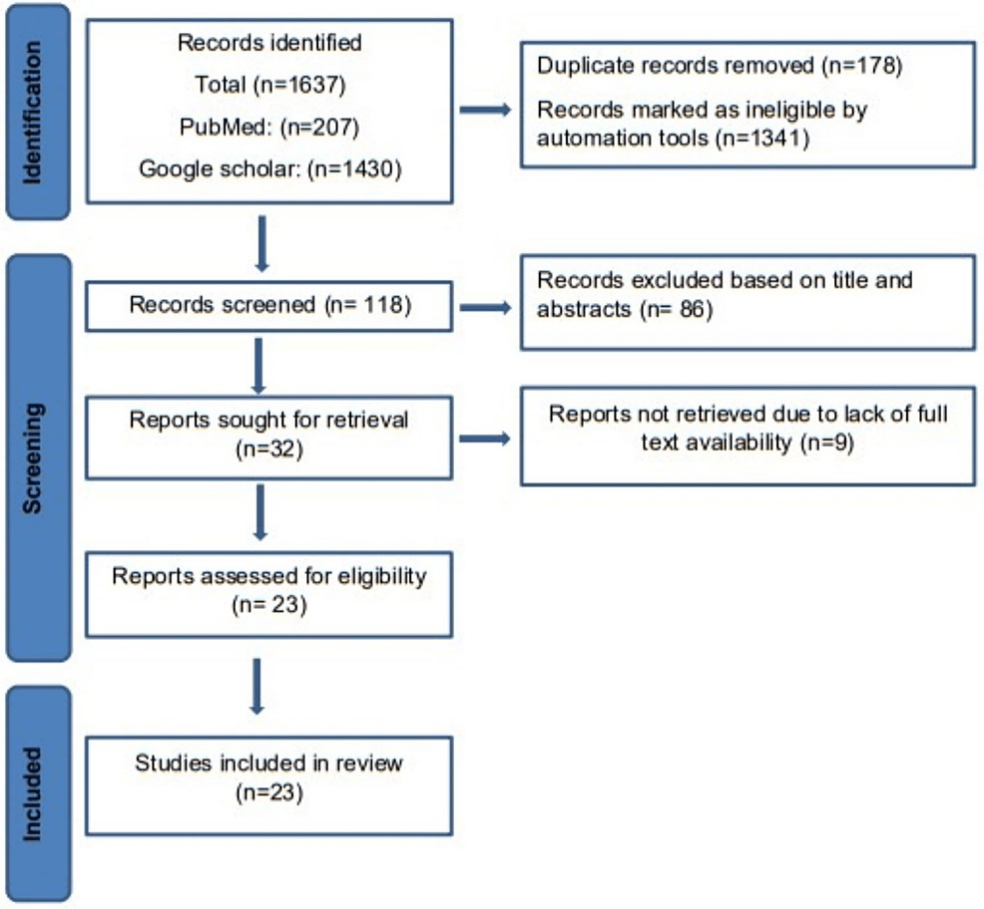
Flowchart of the selection process.

All identified reports were located, recovered, and entered into bibliographic software (Rayyan). Records with duplicates were eliminated. All in vitro studies published in the English language were included in the systematic review. After removing duplicate entries, the published title and abstracts were first evaluated for relevancy using the inclusion and exclusion criteria. The full texts of these pertinent studies were then acquired, reviewed, and ultimately decided to be included in the systematic review. The search process is depicted in Figure 1.

Inclusion Criteria

In vitro studies comparing LAI and UAI and assessing antimicrobial efficiency, smear layer removal, debris removal, or cleanliness on fully formed non-endodontically treated human mature permanent teeth were included, as were publications exclusively in the English language with full text available in hard copy or soft copy.

Exclusion Criteria

Studies that did not use either activation technique; studies that did not assess antimicrobial effectiveness, smear layer removal, debris removal, or cleanliness; laboratory studies using resin models, bovine root models, and endodontically treated teeth; and animal studies were excluded. Randomized control trials, case reports, reviews, and studies unrelated to the subject of the current study were also excluded.

Data Extraction and Analysis

Two reviewers each extracted the data independently. Mutual evaluation of the first 30 articles served as the calibration process. Initial screening was done on all titles and abstracts using the inclusion and exclusion criteria. Subsequently, complete texts of these selected studies were acquired for a second round of screening. To resolve any disagreements, reviewers got together for a meeting and discussion. Studies that passed the second round of screening were sent in for data extraction. The data gathered included the year of study, author, sample size, master apical file, irrigation instrument, wavelength, time, irrigant used, evaluation method, evaluation criteria, presence or absence of isthmus, and results. All 23 relevant articles were suitable for the systematic review (Table 1) [9,13,23-43].

**Table 1:** Studies that fulfilled the inclusion criteria for the systematic review. MAF = master apical file; Er,Cr:YSGG = erbium, chromium-doped yttrium-scandium-gallium and garnet; NaOCl = sodium hypochlorite; LAI = laser-activated irrigation; PUI = passive ultrasonic irrigation; Er:YAG = erbium-doped yttrium aluminum garnet; EDTA = ethylenediamine tetraacetic acid; UAI = ultrasonic-activated irrigation; PIPS = photon-induced photoacoustic streaming; Nd:YAG = neodymium-doped yttrium aluminum garnet; SEM = scanning electron microscope; CFU = colony-forming units; WL = working length; CLSM = confocal laser scanning microscope; SWEEPS = shockwave-enhanced emission photoacoustic streaming

			LAI			UAI						
Author/year	Sample size	MAF	Irrigation instrument	Wavelength	Time	Irrigation instrument	Time	Irrigant	Evaluation method	Evaluation criteria	Isthmus	Results
De Moor, et al., 2009 [23]	70 maxillary canines	#40/.06	Endodontic fiber	Er,Cr:YSGG laser	20s	Stainless steel non-cutting wire (# 20)	20 s	2.5% NaOCl	Groove and hole model	Dentin debris	No	LAI resulted in significantly less debris than PUI
De Groot, et al., 2009 [24]	-	#35/.06	Optical fiber laser tip	Er:YAG 2,940 nm	20s	Stainless steel non-cutting wire (#9 20)	20 s	2% NaOCl	Groove and hole model	Dentin debris	No	The debris score in the LAI group was significantly lower than the PUI group
De Moor, et al., 2010 [25]	100 maxillary canines	#40/.06	Endodontic fiber	Er,Cr:YSGG laser Er:YAG laser	20 s 20 s	Stainless steel non-cutting wire (#20)	20 s 60 s	2.5% NaOCl 2.5% NaOCl	Groove and hole model	Dentin debris	No	ER:YAG laser resulted in less debris score
Peters, et al., 2011 [13]	70 mandibular premolars	#20/.07	Endodontic fiber	Er:YAG laser 2,940 nm	30s	Non-cutting insert	30 s	6% NaOCl	Microbiological analysis and histological analyses	Bacterial counts. Biofilm/Necrotic tissue	No	Laser-activated disinfection was superior
Peeters, et al., 2011 [26]	40 mandibular premolars	#30/.02, #30/.02, #20/.02, #30/.02	Plain fiber (quartz) tip	Er,Cr:YSGG	60 s, 30 s, 60 s	Stainless steel non-cutting wire (#20)	60 s	17% EDTA	SEM	Smear layer, debris	No	A significant difference was found between the smear layer and debris scores for the laser 1 group and those for the UAI, laser 2, and laser 3 groups. Completely clean root canals were found in the laser 1 group
Bago Juric, et al., 2014 [27]	100 mandibular incisors and maxillary second premolars	#30/.06	Endodontic radial firing tip	Er,Cr:YSGG laser 2,780 nm	20 s	Stainless steel 15 K-type file	60 s	2.5% NaOCl	Bacteriologic evaluation	CFUs	No	No differences were noted among the active irrigation techniques
Deleu, et al., 2015 [28]	25 maxillary canines	#30/.06	Plain fiber tip (5 mm from WL), conical PIPS fiber tip (4 mm in the canal), plain fiber tip (2 mm from WL and moved in an up and down motion)	Er:YAG laser 2,940 nm; Er:YAG laser 2,940 nm; diode laser 980 nm	20 s, 20 s, 18 s	Non-cutting #20 file	20 s	2.5% NaOCl	Groove and hole model	Dentin debris	No	The Er:YAG with the plain fiber tip was more efficient than the diode, and Er:YAG laser with the PIPS tip, but the amount of debris was not statistically different from that found in the PUI group. No statistically significant differences were observed between PUI and Er-PIPS groups
Akyuz Ekim, et al., 2015 [29]	80 maxillary centrals	#40/.06	flexible laser fiber tip	Diode laser, 810 nm, Nd:YAG laser, Er:YAG laser 2,940 nm, Er:YAG laser-PIPS 2,940 nm	20s	Stainless steel ultrasonic tip	20 s	2.5% NaOCl, 17% EDTA	SEM	Smear layer	No	PIPS showed the best removal of the smear layer when compared with PUI, Nd:YAG, and Er:YAG, but the difference was not statistically significant. A statistically significant difference was found between the PIPS and diode laser
Sahar-Helft, et al., 2015 [30]	60 single-rooted teeth	#30/.06	Plain-ended sapphire tip (1 mm short of the WL). Plain-ended sapphire tip (upper coronal third)	Er:YAG laser 2,940 nm, Er:YAG laser 2,940 nm	60 s, 60 s	#25/.00 (1 mm short of WL) #25/.00 (upper coronal third)	60 s, 60 s	17% EDTA	SEM	Smear layer	No	The smear layer was removed most efficiently from the entire root canal surface using LAI at low energy with 17% EDTA, inserted either at the working length or only in the coronal upper third of the root. PUI combined with 17% EDTA was found to be depth-dependent
Neelakantan, et al., 2015 [31]	280 mandibular premolar	#25/.06	Plain-ended fiber. Endodontic conical fiber tip	Diode laser, 940 nm, Er:YAG laser 2,940 nm	30 s, 30 s	Ultrasonic files	30 s	3% NaOCl, 17% EDTA	CLSM analysis	Biomass within the dentinal tubules	No	No significant difference between the diode laser and Er:YAG laser. Both diode and Er:YAG laser were more effective than ultrasonic activation
Ayranci, et al., 2016 [32]	48 central incisors	#40/.06	Endodontic conical fiber tip	Er:YAG laser 2,940 nm	60 s, 60 s	Smooth ultrasonic file (15/02).	60 s, 60 s	2.5% NaOCl, 17% EDTA, 2.5% NaOCl	SEM	Smear layer	No	LAI in the pulp chamber with the combination of 2.5% NaOCl and 17% EDTA better removed the smear layer than LAI applied similarly but without EDTA or PUI with the same NaOCl and EDTA combinations using an ultrasonically activated file inserted 1 mm short of the working length
Cheng, et al., 2017 [33]	115 teeth with straight root canals	40#/.04	Optical tip (PIPS)	Er:YAG laser, Er:YAG laser	30 s, 30 s	#25 K-type nickel-titanium file	60 s, 60 s	Normal saline 5.25% NaOCl	SEM	Biofilm, smear layer	No	Er:YAG + NaOCl completely removed the E. faecalis biofilm from the root canal wall and made it the cleanest and most smooth surface among the treatment groups
Kamaci, et al., 2017 [34]	Seventy-five maxillary and mandibular canine teeth	#50/.05	Plain fiber tip (2 mm short of WL). Plain fiber tip (canal orifice) fiber PIPS tip	Diode laser, 980 nm, diode laser, 980 nm Er:YAG laser, 2,940 nm	20 s, 20 s, 20 s	Stainless steel ultrasonic tip (#20)	20 s	2.5% NaOCl	Groove and hole method	Dentin debris	No	No statistically significant difference was noted between the laser groups. UAI removed less debris than the laser groups
Verstraeten, et al., 2017 [35]	Sixty-nine mandibular molars	#30/.09, #30/.07	Plain fiber tip. Conical PIPS fiber tip	Er:YAG laser, 2,940 nm, Er:YAG laser	60 s, 60 s	Non-cutting stainless steel wire (#20)	60 s	2.5% NaOCl	Micro-CT analysis	Dentin debris	Yes	No statistically significant differences were noted in the percentage of debris after irrigant activation between groups were observed
Mancini, et al., 2018 [36]	80 mandibular premolars	#35	Plain fiber tip	Er:YAG laser, 2,940 nm	20 s	#15k fie	1 min	5.25% NaOCl	FESEM	Smear layer	No	LAI showed poor results at 1, 3, and 5 mm from the apex
Donmez Ozkan, et al., 2018 [37]	50 mandibular premolars	#30/.07	PIPS optical fiber tip	Er:YAG laser, 2,940 nm	20s	Ultrasonic tip #15/.02	60 s	5.25% NaOCl	Protein testing model	Biomolecular film	No	PIPS (LAI) method removed more artificial collagen than UAI
Passalidou, et al., 2018 [38]	50 mandibular molars	#25	Endodontic fiber tip 400 µm (canal entrance). Endodontic fiber tip 600 µm (pulp chamber)	Er:YAG laser, 2,940 nm, Er:YAG laser, 2,940 nm	60 s, 60 s	Non-cutting #20 file	60 s	2.5% NaOCl	Images of the sections were analyzed using imaging software	Dentin debris	Yes	The greatest debris reductions were obtained with the LAI protocols
Hage, et al., 2019 [39]	44 mandibular premolar	#25	Conical PIPS tip	Er:YAG laser, 2,940 nm	90 s	#15/0.2	90 s	5.25% NaOCl	Bacteriologic evaluation	CFUs	No	No significant difference was found between PIPS and EndoUltra(UAI)
Race, et al., 2019 [40]	76 mandibular first and second molars	#35/06	Endodontic laser tip	Er,Cr:YSSG laser	90 s	# 20 SATELEC IrriSafeTM file	90 s	15% EDTAC and 4% NaOCl	Bacteriologic evaluation	CFUs	Yes	No significant differences were found between the experimental groups
Betancourt, et al., 2020 [9]	72 single-rooted tooth	#55/.02	Endodontic fiber tip	Er,Cr:YSGG-pulsed laser, 2,780 nm	60 s, 60 s	Non-cutting ultrasonic tip # 25/.00	60 s, 60 s	0.5% NaOCl saline	SEM	CFUs	No	Both agitation techniques LAI and PUI reduced the number of CFUs. Moreover, LAI +0.5% NaOCl and the rest of the groups differed significantly
Yang, et al., 2020 [41]	30 mandibular first and second molars	#30 #35	Pips optical fiber tip. SWEEPS special fiber tip	Er:YAG laser, 2,940 nm, Er:YAG laser, 2,940 nm	90 s, 90 s	#15/.02 ultrasonic tip	90 s	1% NaOCl	Micro-CT evaluation	Dentin debris	Yes	Mesial canals: a significant difference was found between PIPS (58.79%) and SWEEPS (84.31%) and between UAI (50.27%) and SWEEPS. A significant difference was also observed between the PIPS and UAI groups. Distal canals: a significant difference was found between PIPS and SWEEPS and between UAI and SWEEPS. No significant difference was found between the PIPS and UAI groups
Kurzmann, et al., 2020 [42]	80 maxillary canines	#40/.06	Conical PIPS tip	Er:YAG laser, 2,940 nm	20 s, 2 × 20 s, 3 × 20 s, 1 × 20 s, 2 × 30 s, 1 × 60 s	Non-cutting # 20 Irrisafe	60 s	Distilled water	Operating microscope	Dentin debris	No	No statistically significant differences were found between UAI and each individual laser activation technique. No statistically significant differences were found between the X-Pulse tip and the PIPS tip
Mancini, et al., 2021 [43]	85 mandibular premolars	#25/.06	PIPS fiber tip. SWEEPS fiber tip	Er:YAG laser, 2,940 nm, Er:YAG laser, 2,940 nm	3min,30 s	15/.02 tip	3 min, 30 s	17% EDTA, distilled water, 5.25% NaOCl	FESEM	Smear layer	No	PIPS and SWEEPS obtained better results, while only PIPS was superior to PUI in terms of cleanliness

Results

Systematic Review

The search resulted in 1,637 articles. Eliminating duplicates and non-relevant articles resulted in 118 articles. After screening their titles and abstracts, 86 studies were excluded. In total, 32 articles were considered relevant and searched for full-text availability. Finally, the full texts of 23 articles were procured and studied in detail. After studying the full text of these 23 articles, all studies qualified for this systematic review [9,13,23-43].

*Study* *Characteristics*

The 23 studies included in this systematic review were published between 1997 and 2021. Of these, four were published in the 2020s [9,41-43], 17 were published in the 2010s [13,25-40], and two were published in the 2000s [23,24]. Experimental in vitro studies were the focus of all included articles [9,13,23,43]. There were no in vivo studies. The majority of these studies were conducted in hospitals or other academic settings. The median sample size across all reports was 72.

Antimicrobial efficacy was the only outcome of the study in seven reports [9,13,27,31,37,39,40]. Smear layer removal was the only outcome of the investigation in five reports [29,30,32,36,43]. Debris removal was the only outcome of the investigation in nine reports [23-25,28,34,35,38,41,42]. Both antimicrobial efficacy and smear layer removal were the outcomes of investigation in one report [33]. Both smearing layer removal and debris removal were the outcomes of an investigation in one report [26].

Outcome Assessment

Antimicrobial efficacy: Antimicrobial efficacy was discussed in eight out of the 23 studies that were chosen [9,13,27,31,33, 37,39,40]. Of these, five studies claimed that LAI was superior to UAI [9,13,31,33,37]. Despite the fact that the other three studies claimed there was no discernible difference between the two techniques [27,32,33].

Smear layer removal: Seven of the 23 chosen studies [26,29,30,32,33,36,43] discussed the effectiveness of smear layer removal. Six of these studies claimed that LAI was superior to UAI [26,29,30,32,33,43]. The final study found that UAI performed better than LAI [36].

Dentin debris removal: Ten of the 23 papers that were chosen discussed the effectiveness of removing dentin debris [23-26,28,34,35,38,41,42]. Of these, six studies claimed that LAI was superior to UAI [23-26,34,41]. Despite the fact that the other four studies found no appreciable distinction between the two approaches [28,35,38,42].

Discussion

During endodontic therapy, it might be challenging to completely remove biofilms, pathogenic organisms, necrotic tissue, and hard tissue debris from the root canal complex. Because of the complicated structure of root canal architecture, it is hard to reach every area, leaving some unattended. The root canal abnormalities, fins, and isthmuses are filled with a smear layer caused by mechanical instrumentation, which compromises the effectiveness of cleaning and disinfection [44]. Irrigation plays a vital role in cleaning and disinfecting the root canal complex, including fins and isthmuses [45].

In this study, the effectiveness of removing biofilm, smear layer, and dentin debris from the root canal system using LAI and UAI was compared. The results show that irrigation with laser activation is superior to irrigation with ultrasonic activation. However, a moderate level of evidence showed no distinction between irrigation that was activated by a laser and irrigation that was activated by ultrasonic.

As a novel method of agitating intracanal disinfectants, LAI has recently gained popularity. Its result causes cavitation. By powerfully assimilating the laser energy, laser radiation causes temporary cavitation in the irrigant via optical breakdown [46,47]. A well-known method of LAI is photon-induced photoacoustic streaming (PIPS), which uses a fiber tip to pulse at incredibly low energies to convey energy into the solution while only slightly raising dental temperature [48]. It intensifies fluid exchange and the removal of debris by producing vapor bubbles with secondary cavitation effects [17,24]. In clinical use, UAI is undoubtedly the most common method of irrigant activation. Its principal cleaning action is accredited to acoustic microstreaming [49]. In prepared canals, acoustic microstreaming can produce sufficient shear forces to clear the debris.

The study approaches used in the included papers showed considerable variation. The authors demonstrated the methodological differences in the master apical file (MAF), irrigation time, irrigant, and irrigant concentrations after reviewing the included articles. As a result, depending on these factors, the effectiveness of these two strategies may change. It is significant to remember that these variables may affect the findings of the investigation. It is important to stress that the authors of the included research used variable laser parameters with various irrigants, as well as their concentrations and durations of action (Table 1). There were noticeable differences in the ultrasonic devices and their power settings, ultrasonic tips, and irrigation times in the included studies. These variations may have led to conflicting evidence in some comparisons.

This review had certain restrictions. The diversity of the studies included was the first drawback. Therefore, it was inappropriate to perform a meta-analysis [50]. The second drawback was that in vitro research was used to obtain the results of the included studies. A well-designed experimental in vitro investigation, however, may also result in effective treatments for medical issues. The lack of standardized assessment criteria for the evaluation of debris remains and subsequent cleanliness made cross-study comparisons problematic due to the varied evaluation procedures utilized in the investigations.

To conclude, out of the 23 publications that were included, more articles favored LAI in terms of antibacterial effectiveness, smear layer removal, and dentin debris removal from the root canal system. The results of this analysis are relevant to clinical practice because irrigation is a necessary method for removing biofilm, smear layer, and dentin debris from the root canal system during endodontic treatments.

## Conclusions

Within the confines of this systematic review, the current evidence mandates that LAI has superior efficacy over UAI in the elimination of microorganisms, dentin debris, and smear layer from the root canal system. Studies of superior methodologic standards are needed to examine the effectiveness of LAI and UAI for the removal of microorganisms, dentin debris, and smear layer from the root canal system as the results were expressed from moderately high-standard studies.

## Figures and Tables

**Figure 2:**
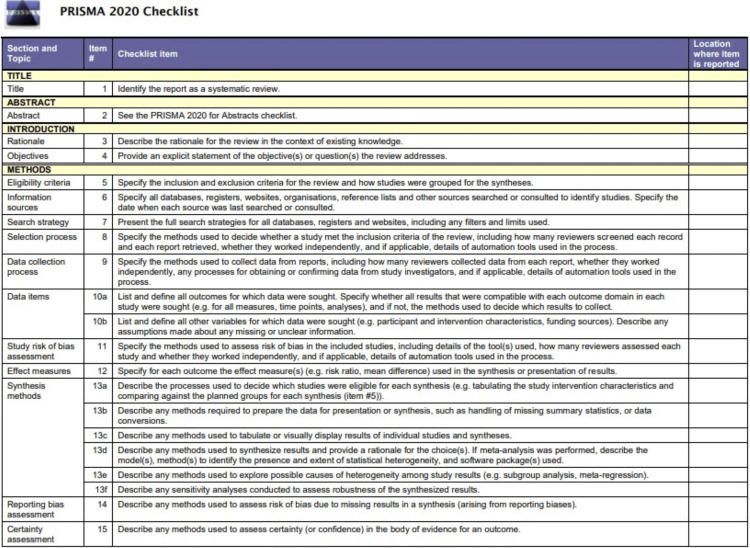
Preferred Reporting Items for Systematic Reviews and Meta-Analyses checklist.

**Figure 3:**
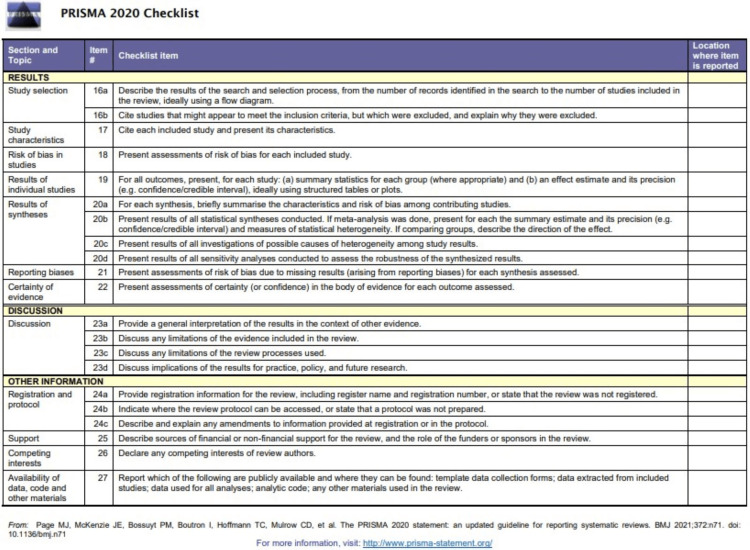
Preferred Reporting Items for Systematic Reviews and Meta-Analyses checklist.
